# Towards a definition of male partner involvement in the prevention of mother-to-child transmission of HIV in Uganda: a pragmatic grounded theory approach

**DOI:** 10.1186/s12913-019-4401-x

**Published:** 2019-08-09

**Authors:** Patience A. Muwanguzi, Louise K. Nassuna, Joachim G. Voss, Joanita Kigozi, Alex Muganzi, Tom Denis Ngabirano, Nelson Sewankambo, Damalie Nakanjako

**Affiliations:** 1 Department of Nursing, School of Health Sciences, Makerere University College of Health Sciences, Kampala, Uganda; 2 Infectious Diseases Institute, Makerere University, Kampala, Uganda; 3 Frances Payne Bolton School of Nursing, Case Western Reserve University, Cleveland, OH USA; 4 School of Medicine, College of Health Sciences, Makerere University, Kampala, Uganda

**Keywords:** Male involvement, PMTCT, Definition, Women HIV, Sub-Saharan Africa

## Abstract

**Background:**

Male partner involvement has been shown to increase mothers’ uptake of Prevention of Mother-to-Child Transmission of HIV (PMTCT) and improve maternal and infant HIV treatment outcomes. Currently, male involvement in PMTCT is measured primarily through men’s attendance at HIV testing and counselling which may not be a true reflection of their engagement. This study therefore set out to explore the meaning of male partner involvement and propose a definition and theoretical model of this concept in PMTCT in Uganda.

**Methods:**

Eight focus group discussions and five in-depth interviews were conducted with couples at three public health facilities and community members in the health facility catchment areas in Uganda. The study employed a grounded theory approach underpinned by the pragmatic philosophical paradigm. Data were analyzed using the constant comparative method, performing three levels of open, axial, and selective coding.

**Results:**

Of the 61 participants, 29 (48%) were male and the majority 39 (63.9%) were in long term marital relationships, while about half were self-employed 29 (47.5%). Three themes emerged for the meaning of male involvement in PMTCT (a) HIV treatment support (b) economic support and (c) psychosocial support. HIV treatment support included adherence support, couples’ HIV counseling and testing, and clinic attendance during and after pregnancy. Participants expressed that men were engaged in PMTCT when they offered economic support by providing basic needs and finances or when they included their female partners in financial planning for the family. Psychosocial support arose from the female participants who defined male involvement as family support, perceived societal recognition and emotional support. Emotional support also included the absence of harm resulting from women’s disclosure of HIV test results to their male partner.

**Conclusions:**

This study proposes a new definition for male partner involvement in PMTCT in Uganda. The definition extends beyond men’s clinic attendance and HIV testing and counselling. Further research should seek to develop and validate tools to accurately measure male partner involvement as the next step in the development of interventions to improve PMTCT outcomes.

**Electronic supplementary material:**

The online version of this article (10.1186/s12913-019-4401-x) contains supplementary material, which is available to authorized users.

## Background

Uganda has the highest national adult HIV prevalence in the East African region, with an estimated national prevalence rate of 6.2% among men and women ages 15–64 years [1]. Mother-to-child transmission is the most common route of infection for HIV-positive children under 5 yrs. The World Health Organisation (WHO) [2] proposes that with an effective prevention of mother-to-child transmission of HIV (PMTCT) program, vertical transmission can be reduced to below 5%; this would mean virtual elimination of mother-to-child transmission (EMTCT). To achieve EMTCT, HIV-positive women and their babies must be enrolled and retained in PMTCT programs and adhere to anti-retroviral therapy (ART) [3]. However, adequate uptake and adherence to these interventions have been challenging for some women if their partners are unaware of or do not support their involvement. For example, some women have refused HIV testing in PMTCT settings because their male partners had either not been present or had not given their permission [4, 5].

In most Sub-Saharan African settings, men control the household resources and often make critical decisions that affect maternal health, including the choice of health services [6]. The WHO thus emphasizes the need to involve male partners in scaling up PMTCT services in Sub-Saharan Africa [3]. Unfortunately, the historic institutionalization of reproductive health as women’s health has contributed to men’s perception of clinic spaces as “women’s spaces” and reproductive health as women’s work. This has produced health services that are not welcoming of men and couples [7–10]. In addition, men often lack information to make informed decisions about the roles they might play in promoting overall family health, including accessing HIV prevention, care and treatment services [11, 12]. It is hypothesized that couple testing may help increase spousal support for women to use PMTCT services, create opportunities for secondary prevention by counseling both men and women about HIV and increasing the identification and treatment of HIV infected persons [11, 13]. It should be noted though that involving men in antenatal care or female reproductive services could have unintended negative consequences such as disempowering women and encouraging relationship disharmony or abuse [14].

According to WHO, male involvement in PMTCT is measured primarily through men’s attendance at HIV testing and counselling and through its associated behavioral and health outcomes (for example, condom usage or adherence to prescribed infant feeding regimens) [11] and antenatal care attendance [15]. In the framework of prevention of mother-to-child transmission (PMTCT), male partner involvement is usually aimed at the health needs of the mother and infant, such as support for mothers’ antenatal HIV testing, uptake of Nevirapine, and formula-feeding or exclusive breastfeeding [14, 16]. Studies have suggested ways to define male partner involvement (MI) and its determinants in a PMTCT setting [17–20]. However, the descriptions focus on clinic attendance and couple counselling and testing [21–23], and in other instances, remain indefinite and vary within different populations and contexts. This imposes challenges in developing focused interventions. Male partner involvement needs to be looked at with a broader lens that involves more than just clinic attendance [18]. Therefore, there is need to have a uniform description of male partner involvement in PMTCT to optimize the development of strategies and interventions that accommodate and enhance male engagement to improve maternal and infant HIV treatment outcomes [17]. This study therefore set out to explore the meaning of male partner involvement and propose a definition and theoretical model of this concept in PMTCT in Uganda.

## Methods

### Study design

This exploratory qualitative study employed the grounded theory approach which is used when a theory is not available to explain a process [24]. In this case, there was no standard definition of male partner involvement in PMTCT [25]. The pragmatic philosophical paradigm underpinned the study. Pragmatism is not committed to any one system of philosophy and reality [26]. This study combined two types of grounded theory by drawing liberally on the reflexive and interpretive nature of the constructivist approach by Charmaz [27] and the more structured systematic approach by Strauss and Corbin [28].

### Study setting

Participants were recruited from three public health facilities and the communities served by these facilities in Kampala district. These health facilities offer HIV services including voluntary counseling and testing (VCT), prevention of mother-to-child transmission (PMTCT), voluntary male medical circumcision (VMMC), provision of anti-retroviral therapy (ART) and the management of certain HIV-related comorbidities [29].

### Sampling strategy

Participants were purposively selected from the public health facilities. However, in keeping with the constructivist approach, subsequent participants were identified by theoretical sampling. “This is a process of data collection in which the researcher simultaneously collects, codes, and analyses data and decides what data to collect next and from whom” [30]. Initially, the researchers collected data from couples at the health facility, but this didn’t capture the views of people who did not go with their partners to the health facility. Therefore, subsequent participants were purposively recruited from the community to highlight those key perspectives. Data saturation determined the number of participants recruited.

### Participant selection

The researchers recruited 61 participants from April to June 2018. The inclusion criterion was willingness to take part in the study. Participants gave individual written consent prior to voluntary participation. All participants were given the choice of participating in either in-depth interviews or focus group discussions and the majority preferred the group discussions.

#### Health facility

Participants recruited at the health facilities were couples attending any health visit from the first trimester of pregnancy up to 18 months after birth. In Uganda, women who attend perinatal care at public health facilities with their partners are given priority for their health provider visit. For this study, the research team approached the couple immediately after they completed their health visit and directed them to a private room. The researchers explained the purpose of the study and sought their consent to take part. The research team invited the couples to either have the interview that day, or to make an appointment to return on another day.

#### Community

Community members received invitations to regular community dialogues through local leaders, announcements in the local media and by word of mouth. A community dialogue is an interactive participatory communication process of sharing information between people or groups of people aimed at reaching a common understanding and workable solution [31]. The research team recruited participants from among community members who attended these dialogues. The dialogues were held to discuss reproductive health with a focus on increasing male partner involvement in all aspects of women’s health. The dialogues were conducted by community health workers. The principal researcher attended all dialogues in the study area for 3 months and shared information about the study. Interested community members received more detailed information privately and selected a date for an in-depth interview or focus group discussion.

### Data collection procedures

The study employed in-depth interviews (IDI’s) and focus group discussions (FGD’s) to collect data for triangulation of methods. The research team conducted IDI’s and FGD’s at the health facility in a private meeting room and in a private secluded area in the community. Each IDI lasted 45 min to 1 hr and partners interviewed individually. The FGD’s had an average of 7–9 people and lasted an hour. Male and female participants took part in separate FGD’s, divided into three age groups (18–25 years, 26–40 years, and above 41 years). All the interviews and discussions were audio recorded with permission.

A moderator and note taker facilitated the FGD’s, and the lead researcher conducted the in-depth interviews. Interview and discussion guides with open-ended and probing questions were used to collect data. (see Additional file 1). Participants decided whether to interview in English or the local language. Data collection was an iterative process in response to evolving study findings. The researchers analyzed the data and field notes daily to decide which group of participants to enroll next and what questions to explore further. This daily analysis also identified emergent categories for the research team to focus on. Participants’ responses determined whether to introduce new topics or adjust the existing question guides [32]. Data collection stopped when no significant new information emerged from interaction with the participants.

### Data management and analysis

Data were transcribed verbatim and transcripts returned to participants for comments and corrections. A language expert translated the approved transcripts into English. The methods proposed by Strauss and Corbin, namely, open, axial and selective axial coding [26, 33] guided manual analysis of the data.

Two researchers independently coded the data, then identified and highlighted concepts along with key phrases and obtained emerging themes. Open coding broke the data down into conceptual components, which brought order and enabled the analysis team to make initial sense of the data. The data from each participant interview and focus group were ‘constantly compared’ for similarities and coherent categories developed from these commonalities. Axial coding linked the various categories and subcategories [34]. The researchers were careful to avoid compressing the data too much in order to keep the richness and distinctiveness of the findings. Selective coding of the transcripts led to the emergence of three core themes to define male partner involvement in PMTCT which were ‘Economic support’, ‘Psychosocial support’ and ‘HIV treatment support’.

All text excerpts were de-identified. A sample of participants provided feedback on the findings to enhance trustworthiness and credibility of data analysis.

## Results

### Participants’ characteristics

Participants were ages 18 to 59 years, the majority 39 (63.9%) were in long term marital relationships and about half of them were self-employed 29 (47.5%). (Table 1).

**Table 1:** Demographics of study participants (*N* = 61)

Participant characteristics	Frequency (Percentage) n (%)
Gender	
Female	32 (52.0)
Male	29 (48.0)
Age range (years)	
18–25	27 (44.3)
26–40	18 (29.5)
Above 41	16 (26.2)
Relationship status	
Long term relationship	39 (63.9)
Divorced/separated	13 (21.3)
Single	9 (14.8)
Employment status	
Self-employed	29 (47.5)
Employed	19 (31.1)
Not employed	13 (21.3)

### Meaning of male involvement in PMTCT

The meaning of male involvement is presented according to the three thematic areas, with the related codes and supporting participant narratives. Table 2 presents the themes and categories and number of responses for each category. (see Additional file 2 for the coding tree).

**Table 2:** Themes and categories of the meaning of male partner involvement in PMTCT

Themes	Subthemes	Number of participants		Total number
		Males *n* = 29	Females *n* = 32	
Economic Support	Provision of needs	25	8	33/61
	Financial provision	29	32	61/61
	Financial planning	0	15	15/61
HIV treatment support	Adherence support	18	6	24/61
	Couple HIV counselling & testing	2	23	25/61
	Clinic attendance	5	13	11/61
Psychosocial support	Family support	7	27	34/61
	Emotional support	1	16	17/61
	Love and belonging	0	8	8/61
	Societal recognition	0	14	14/61

### HIV treatment support

The participants defined key aspects of male partner involvement as HIV treatment support and these included adherence support, couples’ HIV counseling and testing, and clinic attendance.

#### Adherence support

Male partners considered themselves engaged in PMTCT when they reminded their partners to take their medication and give the children their ARV medication. They also felt engaged when they counted their partner’s pills for accountability.

*I remind her to take her pills and sometimes count them just to be sure. I took it as my responsibility to set a pill alarm since I don’t want me or our baby to get infected with HIV.* (IDI with male participant 25 years, serodiscordant relationship).

In addition, some female participants expressed that synchronization of the time of taking their medication may indicate male partner involvement. This is illustrated below;

*… .. when he reminds me to take my medication and when he reminds me about my clinic visits. I would also be happy if we agreed on a specific time to take medication, that way if one of us misses at that time then we can easily remind the other partner.* (IDI with female participant over 22 years, seroconcordant relationship).

#### Couples’ HIV counseling and testing

More women than men thought of couples’ testing as a sign of male partner involvement.

*…*. *the best way is to take a test with the wife that is how I see involvement in HIV. When they receive their results together, then the health worker will inform them of what they are supposed to do, and the man will be more involved and supportive.* (FGD with female participants 26–40 years).

This included testing for HIV together, receiving counseling and testing together and finally regular re-testing for those who were in serodiscordant relationships.

*He should take an HIV test with me and all his other female partners to protect them. I entered the relationship when I was HIV negative but when I came for ANC, I was told that I am HIV positive, when I tell him to come for a test, he isn’t willing. I would feel like he is involved if he takes a test with me.* (IDI with a female participant 38 years, male partner HIV status unknown).

#### Clinic attendance

A few men who defined male involvement as clinical attendance saw it as a way of support and prevention of vertical HIV transmission.

*This is our first pregnancy and I want to be as involved as I can be, so our baby can be born without HIV. The first time we came for antenatal [*antenatal care health visit*], the midwife told me that the baby had a higher chance of being negative if both of us came for clinic visits and continued taking our medications.* (IDI with male participant 25 years).

Many of the male participants did not view pregnancy as a disease and saw no reason to go with their partners for clinic visits. Others were uncomfortable with going to the clinic as illustrated below;

*I keep hearing announcements and my wife tells me that I am supposed to accompany her to the clinic when she is pregnant, but I don’t understand why. She isn’t sick, she can drive herself to the hospital and come back. There is really no reason for me to go there, I can take her to deliver or if she is sick.* (FGD with male participants 26–40 years).

On the other hand, some of the female participants felt that their partners were involved when they escorted them to the clinic and reminded them of their clinic visit days. This also included organizing transport to the clinic and sitting with them at the clinic instead of waiting outside.

*… when he comes with me to the clinic or sometimes when he cannot make it for the visit, he reminds me without fail to go for my visit, organizes transport and he will check to make sure that I have gone.* (FGD with female participants 18–25 years).

*Yes, while coming with me to the clinic is good, he comes and stands outside until it is time for us to go. I can understand because it is not comfortable for him to sit there among all those other women, but he misses out on the information they give us.* (FGD with female participant 26–40 years).

Some participants also wanted their male partners to have their own clinic visits scheduled on the same days as their ANC visits so that they could attend together.

*I feel like he is involved when we do everything related to HIV together during pregnancy. I would be glad if the health workers could help us and make our clinic days fall on the same day, that way we can all receive care as a family.* (IDI with a female participant, 22 years).

### Economic support

Both female and male participants defined male partner involvement in terms of economic support, which comprised provision of needs, financial provision and financial planning.

#### Provision of needs

Male participants expressed that buying food and clothing and providing house rent and money for treatment when their partner or child fell ill was an indication of engagement and support.

*My role in the family is to provide; I feel like I am involved when I meet our family’s needs … these can be in form of food and clothing. When any of them are sick, or when she needs to go for her check-up, I make sure I manage all that.* (FGD with male participants above 41 years).

Some female participants preferred provision of needs over male partner clinic attendance as narrated below.

*He brings me to the hospital and even reminds me to take my medicine [*ART*], but he doesn’t provide anything for me and my child. He doesn’t stay with us, so taking care of this child and myself is not very easy without support. I would prefer that he gives me money to take care of us rather than just taking me to the clinic.* (FGD with female participants 18–25 years).

#### Financial provision

Both female and male participants thought provision of money was a very big part of male partner involvement. This included money for treatment, clinic visits, and for the partner to buy whatever she wanted at her own discretion.

*I make sure I support her by giving her money for the home and I also give her money to use for her own things, no questions asked even though she also earns a salary. I also give her money to pay for all the requirements at the hospital when she goes for her antenatal visits like scan* [Ultrasound scan] *and any blood tests.* (FGD with male participants 26–40 years).

Female participants also expressed a need for money to start a business in order earn their own income.

Some male participants used financial provision to justify their low attendance of clinic visits citing busy work schedules and the fact that they were already providing financially for their partners.

*I work very hard to make sure she is comfortable and has everything she needs. I think that is the highest form of support. Imagine if she goes to the hospital, and they say buy this medication, and she has no money. At least now she can go confidently to any pharmacy.* (FGD with male participants 26–40 years).

#### Financial planning

For some female participants, male involvement meant active engagement in financial planning for the family especially about the future of their children.

*I think also planning together as a family is a way of showing involvement and support. It would be nice if we could plan for the delivery of this child, how we are going to continue living our lives now and how we will use our money as a family for the other older children who are not infected?* (IDI with a female participant, 28 years).

Finally, other participants expressed that they would feel that their male partner was involved, if they were working fewer hours and their male partners took up the bigger percentage of sharing the financial responsibilities.

*I work two jobs now, I must work hard to provide for my children, if my partner were providing more, then I would work less and have less stress. My child has started falling sick more often and I know it is because I am not in position to be with him as much as I should.* (IDI with a female participant, 38 years).

#### Psychosocial support

Psychosocial interventions are interpersonal or informational activities, techniques, or strategies that target biological, behavioral, cognitive, emotional, interpersonal, social, or environmental factors with the aim of improving health functioning and well-being [27]. In this study, psychosocial support comprised family support, emotional support, love and belonging and societal recognition.

#### Family support

Male participants’ defined involvement as helping the partner with childcare and other home chores while she was pregnant.

*When she is pregnant, I always take care of the other children, do some housework, or hire people to help and allow her time to recover and get some rest. I am always conscious of her condition [living with HIV] so I do what I can for her not to overwork herself. (*FGD with male participants 26–40 years*) Sometimes I must stay home with the younger child when she goes to clinic so that she isn’t overwhelmed during her clinic visit.* (FGD with male participants over 41 years).

For the female participants, it was more than just during pregnancy; it was support even after childbirth. This meant making family decisions together, offering help at home, and taking care of the children in her absence to give her time to rest.

*During pregnancy, my husband is very involved and supportive. He will provide everything we need and will help around the house and even help with childcare. However as soon as the baby is born, he mostly gets involved only when the child is sick. I think that involvement should be lifelong especially during the crucial first year when the child can easily turn* [seroconvert]. (FGD with female participants, 26–40 years).

#### Emotional support

Some participants felt that their partners were involved if they supported them emotionally by stopping verbal or physical abuse and talking about HIV and its impacts on their lives.

*I would feel like he is involved if he treated me better. Whenever I go to the clinic for checkup or for treatment, he says so many words* [verbal abuse] *and the time I brought him the invitation letter* [male partner HIV testing invitation letter] *from the clinic he beat me so much. How are we supposed to manage the disease and prevent the children from getting infected when he will not even allow us to discuss this issue which affects both of us?* (FGD with female participants 18–25 years).

It was especially difficult for those living in a serodiscordant relationship, as they felt that their partner blamed them for being HIV positive, and they did not feel supported emotionally.

*I will feel like he is involved in a good way if he doesn’t beat me or always shout at me, it is not my fault that I have HIV. I feel so alone.* (IDI with a female participant, 22 years, serodiscordant relationship).

Emotional support also included, being there for each other and open discussion of the children’s future.

*I feel involved when we talk about HIV and how we can cope with it and make sure the children don’t get infected. Sometimes she gets very discouraged, but I feel like it gets better when we talk about it as a couple.* (IDI with a male participant, 45 years, seroconcordant relationship).

#### Love and belonging

This was expressed by some female participants who were not in long term relationships. They defined one important aspect of male partner involvement as their children finding love and belonging and being accepted by their partner’s family. This included the child meeting the male partner’s extended family, the family accepting and legitimizing the child and even occasionally being acknowledged as one of the legitimate children in the family.

*I will feel like he is involved if he makes sure that the rest of his family know about me and my children especially the one who is HIV positive so that when I die, they will make sure he is taken care of and that he continues taking his medication.* (FGD with female participants, 18–25 years).

#### Societal recognition

the issue of societal recognition was also raised by female participants only; they felt that their male partners were involved when they officially recognized them as their partner in public. This was demonstrated by clinic attendance together or the male partner formally/officially introducing her to his family members.

*I am a second wife, and no one knows about me; to me involvement means an assurance that my child will be well taken care of after I die. This will only happen if people in the community and his family recognize me officially.* (FGD with female participants 18–25 years).

Additionally, some male partners sent their wives to the village when they got pregnant and tested positive for HIV. These women felt such as they were being hidden away from society.

*Involvement means standing with your partner. When I got my HIV test results during the first antenatal visit, he* [male partner] *told me that I should go to the village and wait to have the delivery there*. *I don’t feel like he is supporting me, it seems like he is hiding me away as if he is ashamed of me. He has refused to take the HIV test or to come with me to the clinic. How will I manage to get the medication in the village?* (FGD with female participants 26–40 years).

### Conceptualization and definition of male involvement in PMTCT

**Fig. 1:**
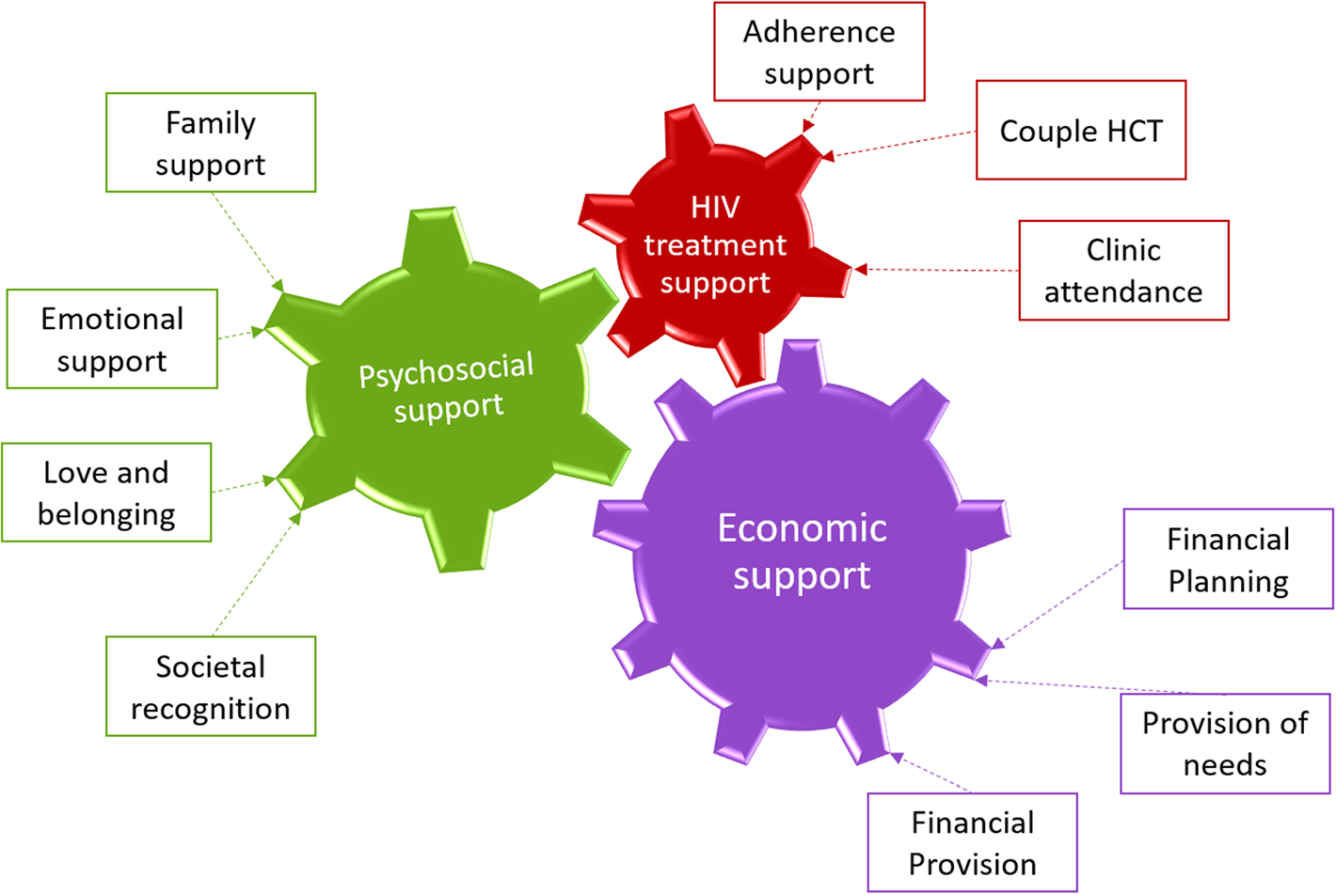
Theoretical model to define male partner involvement in PMTCT

The categories and themes were organized into a theoretical model. The structure of the model is based on the commonalities derived for the meaning of male involvement in PMTCT (Fig. 1). The three themes are presented as cogwheels, a concept derived from physics and engineering. A cogwheel is a rotating machine part having cut teeth, or in the case of a cogwheel, inserted teeth (called cogs), which mesh with another toothed part to transmit torque [35]. Movement of individual cogs on one wheel affects the opposite wheel by increasing speed, increasing force or simply causes motion [36]. Thus, the three themes (cogwheels) of male involvement in PMTCT are interlinked and may positively or negatively influence each other.

Female participants who defined male involvement as psychosocial support expressed that this engagement also influenced economic support as illustrated below;

*Last year my partner of three years allowed the members of his extended family to meet our baby. For me that is the highest level of male involvement because now that the community knows about the child, he is obliged to provide financially for me to be able to take care of the child and of myself. I am now able to take the child for his hospital visits and ensure that he receives proper nutrition and the health worker told me that he is growing normally.* (FGD with female participants 18–25 years).

Another participant expressed that they were able to plan for the family better financially as a couple after they started attending PMTCT together. This implies that HIV treatment support influenced economic support.

*When our daughter was born, I was at the hospital and the midwife talked to me about PMTCT. I never knew that there was so much involved, so now I try to come with her for our baby’s clinic visits. When we go home and discuss what we learned, it allowed us to plan for ourselves and for our daughter’s future. Now when she asks me for money, I know why she needs it.* (FGD with male participants 26–40 years).

On the other hand, some female participants in the community reported that their male partners withdrew financial and psychosocial support, in response to a requirement for HIV treatment support from the health workers. As a result, some participants were unwilling to get their male partners engaged and chose to attend PMTCT activities on their own.

*During my first pregnancy, they gave us letters to give our partners to invite them to come for HIV testing. My husband was so suspicious and wondered why he was being invited for a test. I tried to explain to him that they gave them to all the pregnant irrespective of HIV status, but he wouldn’t listen to me. From that time, our relationship changed, and he doesn’t trust me anymore. He doesn’t beat me and isn’t violent, but we are not the same way we used to be. For this pregnancy I have just decided to completely leave him out of anything to do with the hospital.* (FGD with female participants, 26–40 years).

The model also suggests that no aspect is more important than the other. Therefore, a dentition of male involvement in PMTCT that focuses on only one aspect doesn’t describe the phenomenon in its entirety.

Following an analysis and synthesis of the findings, the authors define male involvement in PMTCT as HIV treatment support, economic support, and psychosocial support with the absence of intimate partner violence.

## Discussion

The study aimed to explore the meaning of male partner involvement and propose a definition and theoretical model of this concept in PMTCT in Uganda. Three themes emerged from the participants understanding of male partner involvement which were economic support, HIV treatment support and psychosocial support. HIV treatment support included adherence support, couples’ HIV counseling, and testing and clinic attendance during and after pregnancy. Participants reported that men were engaged in PMTCT when they offered economic support by providing basic needs and finances or when they included their female partners in financial planning. Psychosocial support arose from participants’ definition of male involvement as family support, perceived societal recognition and emotional support. Emotional support also included the absence of harm resulting from women’s disclosure of HIV test results to their male partner. The participants’ definitions were combined to conceptualize and propose a definition for male partner involvement in PMTCT in Uganda.

The definition proposed in this study complements and takes the discussion forward from recent studies that have suggested ways to describe male involvement in PMTCT. Five of the six items used in the *adhoc* male partner involvement index [10] can be plotted onto two major forms of support proposed in our definition of male partner involvement. (i) The man attends antenatal care with his partner (ii) The man knows the partner’s antenatal appointment, (iii) The man discusses antenatal interventions with his partner and (v) The man has taken time to find out what goes on in the antenatal clinic correspond to ‘HIV treatment support’. While the item (iv) The man supports his partner’s antenatal visits financially corresponds to ‘Economic support’. In Kenya, [20] a previous study defined male partner involvement as both active and passive. Active involvement could include attending ANC/PMTCT appointments at the clinic with his partner. Furthermore, within the household, the man could remind her to take ART medication, remind her of clinic appointments, and follow guidance received from PMTCT service providers (e.g., condom use, consistent feeding practices) [18, 38]. The components of active involvement can be mapped onto the different forms of support in the definition proposed in this study. The most recent study from Malawi [17] classified male partner involvement into positive and negative participation. Positive participation was further categorized into total involvement, reminders, provision and partial involvement. The reminders category can be mapped onto ‘HIV treatment support’ while the provision category can be mapped onto ‘Economic support’ in our definition. Their category of negative participation corresponds to the ‘emotional support’ in our definition where the women considered male involvement as the absence of intimate partner violence (IPV). In a study conducted in Uganda on the challenges faced by women living with HIV during the perinatal period, some of the negative types of involvement included IPV. Others included denial of access to treatment by their partners and refusal of permission to work during pregnancy and the postpartum period [37]. These findings agree with what female participants described as male partner involvement in this study.

Male and female participants had slightly varying perceptions of male partner involvement with the men focusing on the economic aspect while the women’s definition was more holistic. This concurs with findings where male partner involvement focused on provision of resources and reminders of antenatal appointments [17, 18] and saw their direct role as largely financial [12]. Women living with HIV in Uganda reportedly experienced challenges associated with dependence on their partners to satisfy basic needs while navigating polygamy and multiple partnerships [38]. Most of the women in this study defined male partner involvement largely as psychosocial support. They also preferred male partner involvement throughout the perinatal period because they felt that they still needed help after birth with family support and coping emotionally. This agrees with findings from South Africa [39] where the female partners expressed a desire for their partners to be present during antenatal sessions and delivery. There was an observable difference under the HIV treatment support theme, where men reported that adherence support was the main way they demonstrated involvement in PMTCT. The women on the other hand, defined male partner involvement as couple HIV testing. This agrees with findings where men were not as concerned with getting tested or with prevention of HIV infection from mother to their child [37] .

Based on the study findings, the authors make the following recommendations. Firstly, the definition of male partner involvement needs to be broadened and conceptualized beyond HIV testing during and after pregnancy to include the different aspects of support. Policies on measurement need to be reviewed and a new definition which incorporates community perspectives should be considered. However, before this can be done, measurable indicators, tools and registers need to be designed for correct measurement and documentation.

Secondly, some participants only got engaged in PMTCT after interacting with health workers. Therefore, health workers still have a role to play in sensitizing families about the importance of male involvement on maternal and child health outcomes including synchronizing clinic visits for families. In that regard, PMTCT strategies/policies should focus on couples’ involvement in PMTCT. Findings from Uganda showed that men were less likely to get an HIV test if they accompanied their spouses [37] because they worried that their peers would consider them weak; therefore, the cultural context is important. Finally, the male participants were uncomfortable with the public health facilities because there was not much else for them to do besides taking an HIV test and waiting for their partner. Therefore, integration of male friendly packages during the perinatal period is essential to make men comfortable at public health facilities and increase their engagement in PMTCT.

### Study limitations

Although useful insights can be drawn from the findings of this study, the results may be context specific; however, the methods can be replicated. The researchers interviewed study participants at only one point along the PMTCT cascade. Consequently, it is possible that their recollection and attitudes towards involvement changed over time and the cross-sectional nature of the study does not capture those changes.

## Conclusions

Current indicators for male partner involvement may not accurately reflect what both women and men consider as male partner involvement in PMTCT. We propose further research to develop a scale to measure male partner involvement in the Prevention of Mother-to-Child Transmission of HIV as the next step in developing interventions to improve PMTCT outcomes.

## Additional file

Additional file 1: Focus Group Discussion guide (DOCX 20 kb)

Additional file 2: Coding tree for male partner involvement in PMTCT (DOCX 18 kb)

## Abbreviations

ANC: Antenatal care
ART: Anti-Retroviral Therapy
EMTCT: Elimination of Mother-to-Child Transmission
FGD: Focus Group Discussion
IDI: In-depth interview
IPV: Intimate Partner Violence
MI: Male Involvement
PMTCT: Prevention of Mother-to-Child Transmission of HIV
WHO: World Health Organisation
