# Supplementary material for: Towards a definition of male partner involvement in the prevention of mother-to-child transmission of HIV in Uganda: a pragmatic grounded theory approach
Source: BMC Health Serv Res. 2019 Aug 9;19:557. doi: 10.1186/s12913-019-4401-x (PMC6688339; doi:10.1186/s12913-019-4401-x)
Supplement: Supplementary file 1 — Focus Group Discussion guide (DOCX 20 kb) [file 12913_2019_4401_MOESM1_ESM.docx]

**Additional file 1: Focus Group Discussion Guide**

Thank you for agreeing to participate in this discussion about men’s participation in the prevention of mother-to-child transmission of HIV (PMTCT). This interaction will take about one hour.

| **Date:**  **Start/End Time (if available):** | **Location of FGD:** |
| --- | --- |
| **Focus group discussion facilitator:** |  |
| **Note-taker:** |  |
| **Number of participants in this group (total):** | **Important note for the FGD:**  *Given the discussion topic of these focus groups,*  *it is recommended that age groups should not be mixed i.e. create FGD among the 18-25 years, 26-40 years and above 41 years.* |

1. What do you know about the prevention of mother to child transmission of HIV (PMTCT)?
2. What is the main aim of PMTCT? (Provide information if needed)
3. Why is it important for a pregnant or lactating mother to take ARVs? (Probe; what do you think happens to a person who doesn’t take his/her ARVs as prescribed?)
4. What is the role of men is in ensuring their partner access PMTCT services? Would you access these services (emphasize HIV testing) together with your partner at the facility? (Probe; if yes, and if no as below)
5. If yes, what are the things that will encourage you to come to the facility with your partner? If no, what things would discourage you from accessing services with your partner?
6. What does male involvement in PMTCT mean in your own terms? (Probe: specific examples along PMTCT cascade)
7. Why is it important for men to participate in PMTCT?
8. What could be done to improve men’s participation in PMTCT? (Probe: what things can be done at the facility or at home)?

*If you have any concerns, or think of additional information that should be shared, you can share them with the facility in charges in your catchment area, and these will ensure this information is relayed back to us.*
