# Supplementary material for: Towards a definition of male partner involvement in the prevention of mother-to-child transmission of HIV in Uganda: a pragmatic grounded theory approach
Source: BMC Health Serv Res. 2019 Aug 9;19:557. doi: 10.1186/s12913-019-4401-x (PMC6688339; doi:10.1186/s12913-019-4401-x)
Supplement: Supplementary file 2 — Coding tree for male partner involvement in PMTCT (DOCX 18 kb) [file 12913_2019_4401_MOESM2_ESM.docx]

**Additional file 2: Coding tree for male partner involvement in PMTCT**

| **Categories** | **Broader codes** | **Codes** |
| --- | --- | --- |
| Economic Support | Provision of needs | - Buys food and clothing; - brings weekly supplies; - opens a credit line at the supermarket - brings other sick children to hospital; - pays rent; - pays school fees; - meets survival (*basic*) needs |
|  | Financial provision | - Money for clinic visits; - money for treatment when sick; - leaves money for the week; - gave money to start business; - gives money for ‘women’s things’; - ensures his wife is always smartly (f*ashionably*) dressed |
|  | Financial planning | - Both contributing to the home development; - planning for finances together; - agreement on investments; - equal distribution of money with his other ‘known’ female partner; - gives permission to wife to work fewer hours and he provides |
| HIV treatment support | Adherence support | - Reminder to take pills; - pill counting; - reminder to give child ARVs; - taking ARVs together; - helping to give child ARVs; - accepting to use a condom at home |
|  | Couple HIV counselling and testing | - Testing for HIV together; - testing all other partners in the sexual network for HIV; - getting results together; - regular testing; - sharing results of his other wife |
|  | Clinic attendance | - Escort to clinic; - reminder clinic visit dates; - sit with me at clinic; - make our (*couple*) clinic visits on same day; - coming in not sitting outside clinic; - waiting with me at clinic; - gives permission to attend clinic; - gives money to attend clinic without verbal and physical abuse |
| Psychosocial support | Family support | - Care of other HIV negative children; - helping with family care in my absence; - making family decisions together; - more help in the home; permission to continue working |
|  | Emotional support | - No physical abuse related to HIV status; - talking about the disease; - not telling whole world we are discordant; - coping with side effects of drugs; - discussion of children’s uncertain future |
|  | Love and belonging | - His family acceptance of our child; - My child meeting his family; - His family knowing our children officially - care of my children when I die; - my child recognized by his family |
|  | Societal recognition | - Clinic attendance together; - official formal wedding or function; - meeting male partner’s family members; - spending more nights at home together; - male partner gives permission for her to stay in the city when very sick instead of sending her to the village to die |
